# Emerging roles of mechanosensitive ion channels in acute lung injury/acute respiratory distress syndrome

**DOI:** 10.1186/s12931-022-02303-3

**Published:** 2022-12-20

**Authors:** Qi Jia, Yiyi Yang, Xiangdong Chen, Shanglong Yao, Zhiqiang Hu

**Affiliations:** Department of Anesthesiology, Union Hospital, Tongji Medical College, Huazhong University of Science and Technology, Wuhan, China

**Keywords:** Acute lung injury/acute respiratory distress syndrome, Mechanosensitive ion channels, Epithelial sodium channel, Piezo channels, Transient receptor potential channels, Two-pore domain potassium ion channels

## Abstract

Acute lung injury/acute respiratory distress syndrome (ALI/ARDS) is a devastating respiratory disorder with high rates of mortality and morbidity, but the detailed underlying mechanisms of ALI/ARDS remain largely unknown. Mechanosensitive ion channels (MSCs), including epithelial sodium channel (ENaC), Piezo channels, transient receptor potential channels (TRPs), and two-pore domain potassium ion (K2P) channels, are highly expressed in lung tissues, and the activity of these MSCs can be modulated by mechanical forces (e.g., mechanical ventilation) and other stimuli (e.g., LPS, hyperoxia). Dysfunction of MSCs has been found in various types of ALI/ARDS, and MSCs play a key role in regulating alveolar fluid clearance, alveolar epithelial/endothelial barrier function, the inflammatory response and surfactant secretion in ALI/ARDS lungs. Targeting MSCs exerts therapeutic effects in the treatment of ALI/ARDS. In this review, we summarize the structure and functions of several well-recognized MSCs, the role of MSCs in the pathogenesis of ALI/ARDS and recent advances in the pharmacological and molecular modulation of MSCs in the treatment of ALI/ARDS. According to the current literature, targeting MSCs might be a very promising therapeutic approach against ALI/ARDS.

## Introduction

Acute lung injury (ALI) and its more severe form, acute respiratory distress syndrome (ARDS), are devastating respiratory disorders with high morbidity and mortality rates [1–3]. ALI/ARDS is a consequence of noninfectious (e.g., lung ventilator stretches, trauma and hemorrhage) and infectious (e.g., sepsis, pneumonia, viral infection and pancreatitis) causes [2–5]. The pathogenesis of ALI/ARDS is complex; it is characterized by disruption of the alveolar septal barrier and severe inflammation within the lung, which results in patchy alveolar flooding, excessive immune cell (neutrophil and macrophage) influx, inflammatory cytokine release, impaired surfactant synthesis and significant hypoxemia [1, 3]. The available treatments for ALI/ARDS are limited and include mechanical ventilation (MV) and oxygen supplementation (hyperoxia: HO) [1, 6]. Unfortunately, inappropriate MV (e.g., high tidal volume ventilation) or HO can induce and aggravate lung injury [1, 7–9]. Therefore, understanding the pathological mechanisms of ALI/ARDS and their molecular drivers is crucial for the development of novel therapeutic strategies for ALI/ARDS.

Mechanosensation of the environment is a major determinant of cell fate, which is needed for living organisms to receive and convert mechanical perturbation to electrochemical signals (mechanotransduction) [10, 11]. Numerous molecules, including ion channels, cytoskeletons, focal adhesion-associated molecules and G protein-coupled receptors, mediate the mechanosensation/mechanotransduction process and the response to mechanical forces [12–18]. Among these molecules, mechanosensitive ion channels (MSCs) have been suggested to be the most important for mediating the mechanosensation/mechanotransduction process. Over the past decades, several types of channels, including the epithelial sodium channel/degenerin (ENaC/DEG) family, Piezo channels, transient receptor potential (TRP) superfamily, two-pore domain potassium (K2P) channels, and transmembrane channel-like 1/2 (TMC1/2) channels, have been validated as bona fide MSCs [19–23], and dysfunction of these MSCs is considered to be involved in various pathological conditions and diseases (e.g., pain, cancers, pulmonary hypertension) [24–26].

Cells in lung parenchyma, airways, and pulmonary and bronchial vascular systems are continually subjected to various mechanical forces (e.g., shear stress, stretch, and hydrostatic pressure) associated with lung inflation, vascular perfusion, and physical activity [27, 28]. MSCs (e.g., the ENaC/DEG family, Piezo channels, TRP superfamily, and K2P channels) are expressed throughout lung tissues (e.g., epithelial, endothelial and immune cells) [29–32]. Accumulating evidence suggests that MSCs play a crucial role in the pathogenesis of ALI/ARDS. MSCs are not exclusively activated by mechanical forces (e.g., mechanical stretch and shear stress) but are also modulated by a variety of other stimuli that are altered in ALI/ARDS lungs, including changes in pH or temperature, inflammatory cytokines (e.g., TNF-α), drugs (e.g., volatile anesthetics), biological ligands (e.g., ATP and lipids), and changes in the membrane potential (voltage dependency) [29, 33, 34]. Extensive studies have reported that the expression and/or activity of these MSCs are altered in ALI/ARDS lungs [34–36]. MSCs have also been suggested to play a crucial role in mechanical ventilation, hyperoxia, and infectious- and cytokine-mediated signaling during epithelial, endothelial, and immune cell activation in ALI/ARDS lungs [7, 32, 37].

In this review, we focused on the structure and functions of several well-recognized MSCs, summarized the roles of MSCs in the pathogenesis of ALI/ARDS and described recent advances in the pharmacological and molecular modulation of MSCs in the treatment of ALI/ARDS. Combining our knowledge will undoubtedly unify these fields, and together, the findings suggest that targeting MSCs is a very promising novel therapeutic approach against ALI/ARDS.

## Force transduction and gating mechanisms of MSCs

**Fig. 1:**
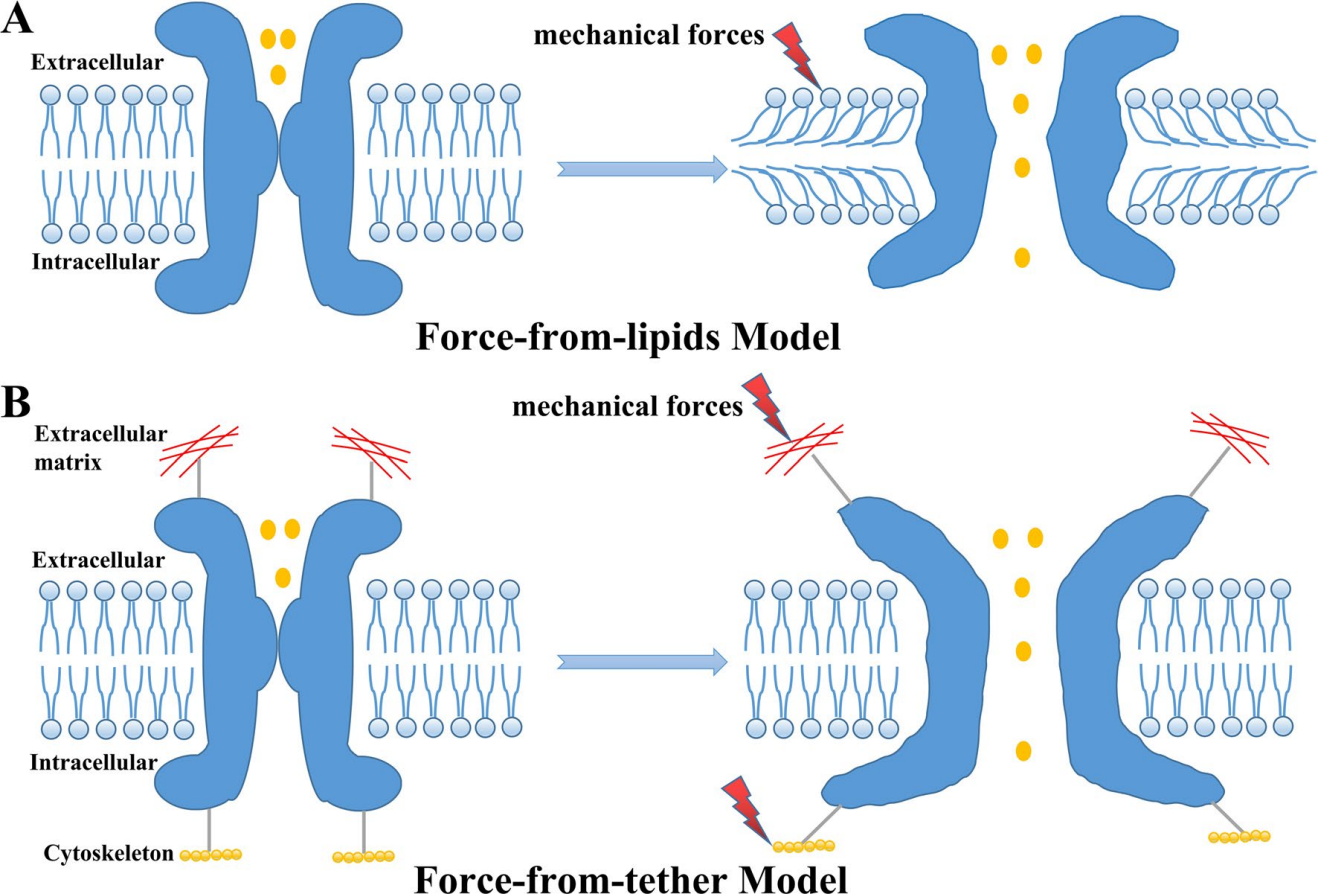
Mechanistic models of mechanosensitive ion channels (MSCs). **A** Force-from-lipids model. Mechanical forces applied to a lipids bilayer can directly activate MSCs without a need for additional elements such as cytoskeleton or accessory proteins. **B** Force-from-tethers model. MSCs are tethered to the extracellular martrix (ECM) and/or the cytoskeleton, and the transmission of mechanical forces through a tether connecting MSCs can change the conformation of MSCs and gate these channels

One central question concerns how mechanical forces in lung tissues are transduced to gate MSCs. Distinguished from other molecules, MSCs can rapidly respond to mechanical forces within milliseconds and convert physical stimuli to electrochemical signals [22, 38]. As illustrated in Fig. 1, two classic mechanogating models have been proposed to explain how mechanical forces can activate MSCs: (1) the “force-from-lipids model” proposes that mechanical forces applied to the lipid bilayer can directly activate MSCs without the need for additional elements such as the cytoskeleton or accessory proteins; and (2) the “force-from-tethers model” proposes that MSCs are tethered to the extracellular matrix (ECM) and/or the cytoskeleton, and the transmission of mechanical forces through a tether connecting MSCs can change the conformation of MSCs and gate these channels [17, 22, 39, 40]. To our knowledge, Piezo channels and several K2P channels have been reported to use the force-from-lipids models to sense mechanical perturbation across a cell [20, 22, 41]. ENaC channels and TRP channels have been elegantly demonstrated to interact with ECM or microtubules in a tethered gating model [19, 21]. Thus, mechanical forces in lung tissues are directly transmitted to gate MSCs through the lipid bilayer or indirectly transmitted to gate MSCs through tethers (e.g., cytoskeleton or ECM) [19, 29, 38], and alternations in the mechanic properties of ALI/ARDS lung tissues, including cytoskeletal remodeling and ECM stiffness, may also influence MSC-mediated mechanotransduction [42, 43].

## Dysregulated mechanotransduction in ALI/ARDS

The lung is an inherently mechanosensory organ. During spontaneous respiration and mechanical ventilation, the lung tissues are subjected to various mechanical forces in the form of shear stress, cyclic stretch, hydrostatic pressure, tension/compression, or various grades of ECM stiffness [27, 44]. MSCs expressed in lung tissues can be activated by mechanical forces within the physiological range and play a key role in regulating lung development, alveolar epithelial/endothelial barrier function and the inflammatory response [31, 45]. However, both pulmonary insult (e.g., ventilator-induced stretch) and extrapulmonary insult (e.g., sepsis) can result in an alteration of lung tissue mechanical properties (e.g., cytoskeletal remodeling and ECM stiffness) [46–48], and excessive mechanical forces induce aberrant activation of these MSCs, which subsequently triggers multiple signaling pathway activation and influence pulmonary and systemic cell dysfunction [7, 33, 42].

**Fig. 2:**
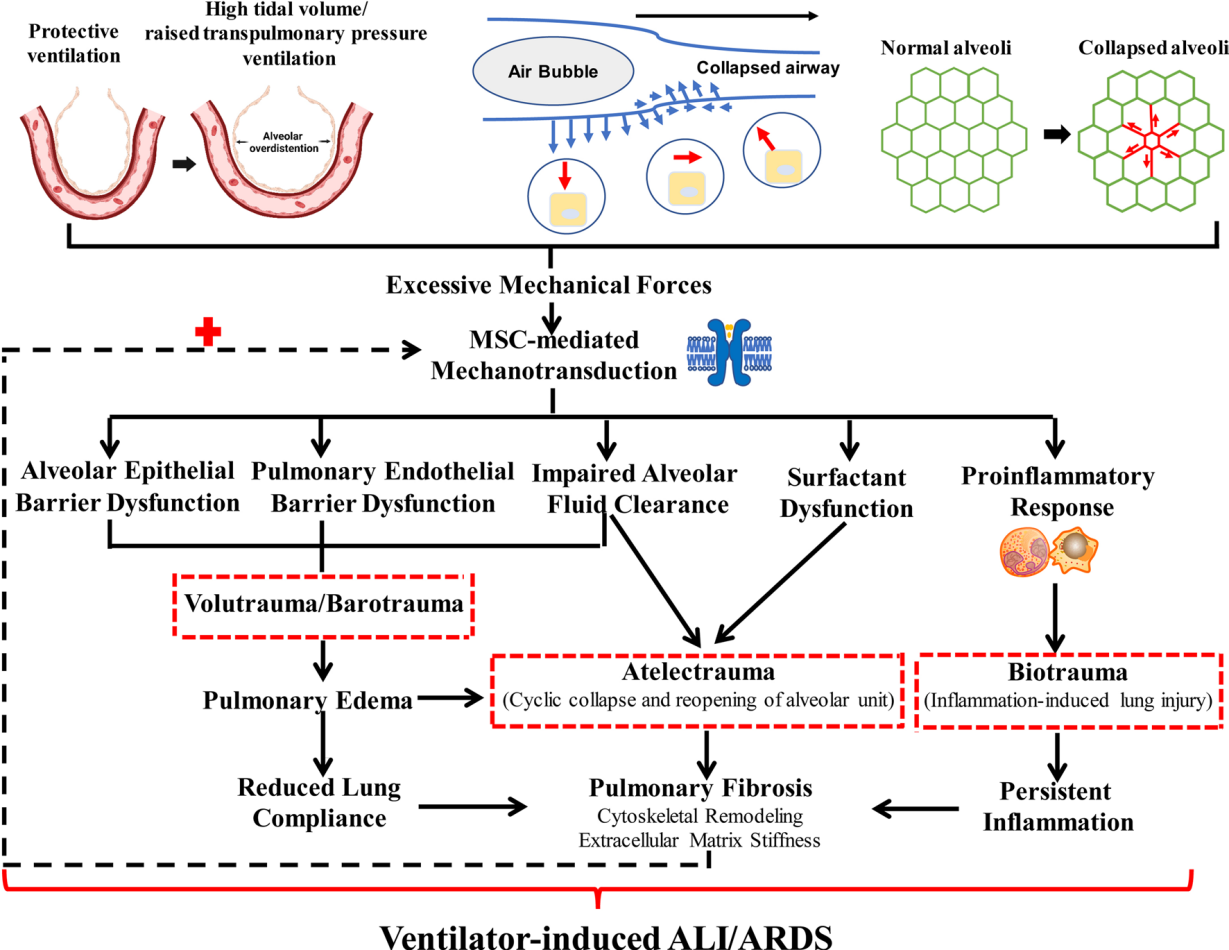
Dysregulation of mechanotransduction in ventilator-induced acute lung injury/acute respiratory distress syndrome (ALI/ARDS). In ventilator-induced ALI/ARDS, excessive mechanical forces induce aberrant activation of mechanosensitive ion channels (MSCs)-induced mechanotransduction, which can lead to volutrauma, barotrauma, impaired alveolar fluid clearance, surfactant dysfunction and a pro-inflammatory response and subsequently causes pulmonary edema, aletectrauma and biotrauma. Furthermore, mechanical forces and persistent inflammation also stimulate stimulate cytoskeletal remodeling and extracellular matrix stiffness, which persist during the fibroproliferative phase of ARDS and ultimately lead to pulmonary fibrosis. The fibrotic lung may further enhance MSC-mediated mechanotransduction and exacerbates the progression of ALI/ARDS

Increasing evidence suggests that dysregulated mechanotransduction may be one of the major contributors to the pathogenesis of ventilator-induced ALI/ARDS (summarized in Fig. 2) [7, 49, 50]. Mechanical ventilation with high tidal volumes or raised transpulmonary pressure can cause volutrauma and barotrauma through alveolar overdistention [51]. Additionally, high mechanical forces generated from cyclic collapse and reopening of atelectatic but recruitable alveolar can also cause lung injury (atelectrauma). For instance, high mechanical forces are generated during recruitment at the interface between the air bolus and collapsed airway and collapsed or flooded alveoli can increase the distention of surrounding alveoli [52]. Excessive mechanical forces (e.g., mechanical stretch) exerted on alveolar units leading to aberrant activation of MSCs (e.g., Piezo1), and aberrant enhancement of MSC-mediated mechanotransduction subsequently activates multiple downstream signaling pathway and results in disruption of alveolar epithelial/endothelial barrier function and pulmonary edema [33, 49]. For instance, excessive mechanical stretch during high tidal volume ventilation induces augmentation of endothelial Piezo1-mediated Ca^2+^ influx, and subsequently increases Ca^2+^-dependent calpain activity and result in pulmonary endothelial hyperpermeability and pulmonary edema [49]. Moreover, dysregulation of MSC-mediated mechanotransduction (e.g., ENaC, TRPV4 and Piezo1) also impairs alveolar fluid clearance (AFC) and causes surfactant dysfunction [53, 54], which may aggravate regional atelectasis. Furthermore, aberrant activation of MSCs (e.g., TRPV4) by excessive mechanical forces also triggers numerous cellular signaling pathways, including activation of a pro-inflammatory and pro-injurious cytokine cascade [55]. This cascade termed biotrauma may exacerbate injury even in alveolar units not faced with significant mechanical insult [51, 56]. Moreover, mechanical forces and persistent inflammation also stimulate cytoskeletal remodeling (e.g., microtubule disassembly), provisional ECM formation and stiffness [46, 47, 57], which persist during the fibroproliferative phase of ARDS and ultimately lead to pulmonary fibrosis. As demonstrated by recent studies, the activities of MSCs (e.g., TRPV4) are enhanced by increasing stiffness of fibrotic and inflamed lung tissues [42, 58], thus the fibrotic lung may further enhance MSC-mediated mechanotransduction and exacerbate the progression of ALI/ARDS.

**Fig. 3:**
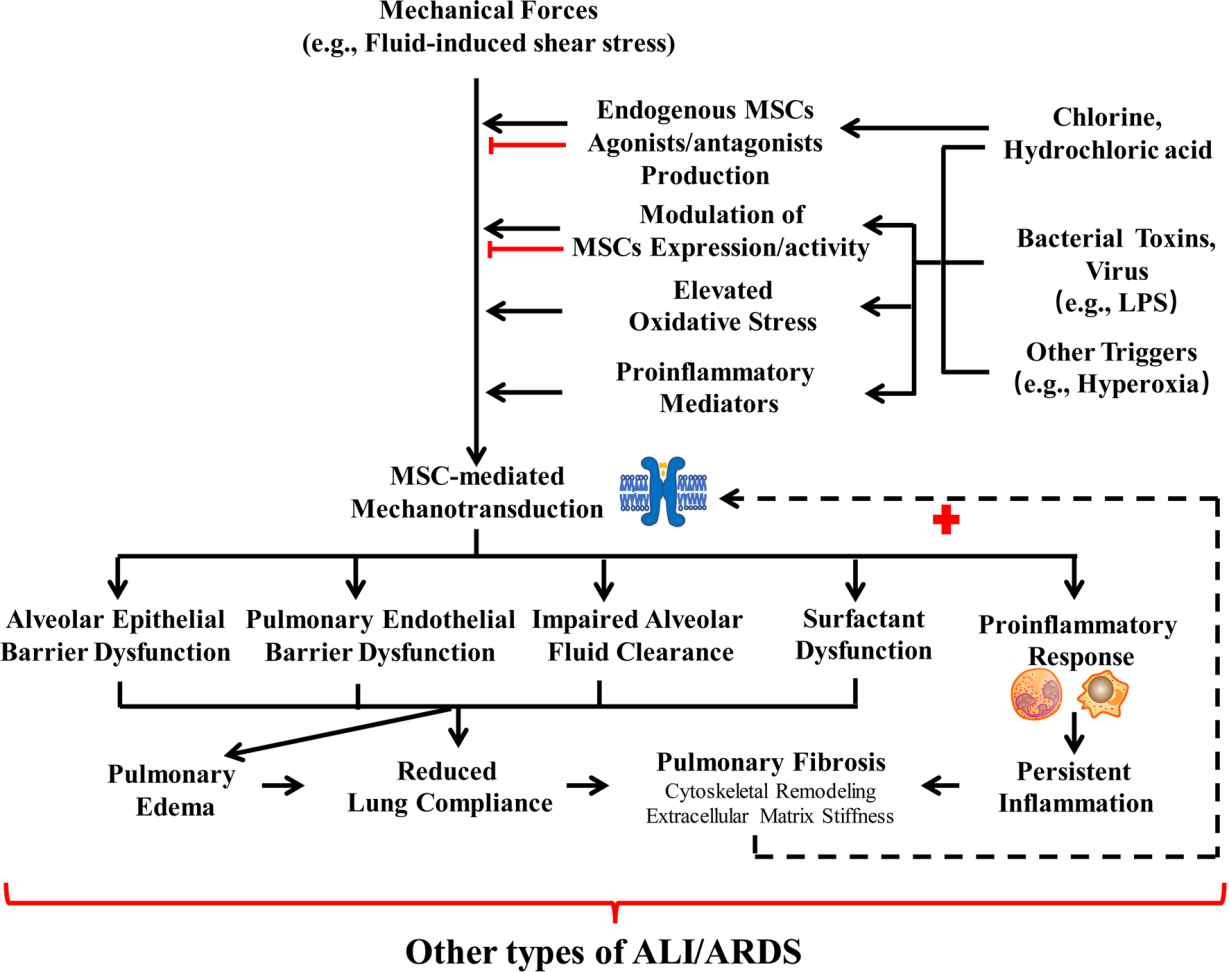
Dysregulation of mechanotransduction in other types of acute lung injury/acute respiratory distress syndrome (ALI/ARDS). In chemical (e.g., chlorine, hydrochloric acid)-induced ALI/ARDS, chemical stimuli can induce the production of several endogenous mechanosensitive ion channels (MSCs) agonists/antagonists, which can affect MSC-mediated mechanotransduction. Furthermore, both chemical stimuli, such as bacterial toxins (e.g., LPS) and viruses, and other stimuli (e.g., hyperoxia) can modulate the expressions and/or activities of MSCs, elevate oxidative stress and increase the production of proinflamatory mediators, which result in the dysregulation of MSC-mediated mechanotransduction. Dysregulation of mechanotransduction can cause alveolar epithelial/endothelial barrier dysfunction, impaired alveolar fluid clearance, surfactant dysfunction, a pro-inflammatory response, and pulmonary edema. Furthermore, persistent inflammation also stimulates cytoskeletal remodeling and extracellular matrix stiffness, which persist during the fibroproliferative phase of ARDS and ultimately lead to pulmonary fibrosis. The fibrotic lung may further enhance MSC-mediated mechanotransduction and exacerbates the progression of ALI/ARDS

Moreover, dysregulation of MSC-mediated mechanotransduction is also suggested to be involved in other types of ALI/ARDS (summarized in Fig. 3) [37, 59]. In fluid-induced ALI/ARDS, high shear stress generated from rapid administration of intravenous 0.9% saline solution are suggested to induce TRPV4 overactivation and subsequently result in pulmonary endothelial hyperpermeability and pulmonary edema, and fluid-induced pulmonary edema can be alleviated by knockout of TRPV4 or treatment with TRPV4 inhibitor [59]. In addition to mechanical forces, MSC-mediated mechanotransduction can also be affected by other stimuli (e.g., chemical stimuli and bacterial toxins). For instance, in chemical-induced ALI/ARDS, several chemical stimuli (e.g., hydrochloric acid and chlorine) increase the production of endogenous TRPV4 agonists (e.g., *N*-acylamides) which can excessively activate TRPV4 and induce pulmonary edema, protein leakage, and immune cell filtration in the lungs, but the exact cell-type contributing to the effect of TRPV4 are needed further investigation [37]. In sepsis-induced ALI/ARDS, MSCs (e.g., TRPV4) can be directly activated by several bacterial toxins (e.g., LPS) and indirectly activated by elevated oxidative stress (e.g., ROS) and pro-inflammatory mediators, and dysregulation of MSC-mediated mechanotransduction contribute to the pathogenesis (e.g., disruption of alveolar epithelia/endothelial barrier function) of sepsis-induce ALI/ARDS [42, 60]. Similar to mechanical forces, both other pulmonary insults (e.g., hydrochloric acid, hyperoxia) and extrapulmonary insults (e.g., sepsis) can also promote the fibroproliferative response (e.g., ECM stiffness, cytoskeletal remodeling) in ALI/ARDS [61–63], which may further enhance the activities of MSCs and exacerbate lung injury [42, 64].

## MSCs in ALI/ARDS

### ENaC in ALI/ARDS

The epithelial sodium channel/degenerin (ENaC/DEG) superfamily are voltage-insensitive but Na^+^-selective channels, including vertebrate ENaC and acid-sensitive channels (ASICs), nematode DEGs, drosophila pickpocket (PPK) and ripped pocket (RPK), and peptide gated Hydra sodium channels (HyNaCs) [17]. In vertebrates, ENaC has been confirmed to be an MSC consisting of α, β, and γ subunits, and its activity can be regulated by mechanical forces (e.g., shear force) [65]. A recent study found that glycosylated asparagines and their *N*-glycans are part of tethers for the mechanical activation of ENaC by shear force (the force-from-tethers model) [19]. ENaC is mainly expressed in epithelial tissues such as the kidney, lung, and colon and plays a key role in maintaining electrolyte and fluid homeostasis [66–68]. In the lung, ENaC is highly expressed in the apical membrane of alveolar epithelial type I and type II cells, and together with the basolaterally expressed Na^+^-K^+^-ATPase, serves as the main force driving Na^+^ transepithelial reabsorption to drive fluid out of alveolar spaces [67, 69, 70]. Lung apical ENaC can be activated in response to the application of laminar shear stress to alveolar epithelial cells, and elevated laminar shear stress can increase the ENaC open probability and accelerate alveolar fluid clearance [38, 71].

Pulmonary edema is an important pathological feature of ALI/ARDS and is characterized by impaired AFC and alveolar-capillary hyperpermeability [67, 72]. Impaired AFC is associated with higher mortality in ALI/ARDS patients, and improving the AFC capacity is necessary for the resolution of ALI/ARDS [72]. ENaC is thought to be the rate-limiting factor for AFC during pulmonary edema, and mice lacking the α, β and γ subunits are unable to clear edema fluid and die shortly after birth with flooded lungs [73, 74]. Since ENaC can be directly activated by shear stress, elevated shear stress generated from enforced ventilation increases the activity of ENaC and improve AFC capacity [38], which may be a protective mechanism to counteract the pulmonary edema in ventilator-induced ALI/ARDS. Moreover, extensive studies have shown that inhibition of ENaC contributes to the impaired AFC of ALI/ARDS. In chemical gas-induced ALI/ARDS, chemical gas (e.g., Cl_2_) increases the plasma levels of cell-free heme, which can bind to ENaC and inhibit its activity, and these steps impair AFC capacity and cause alveolar-capillary hyperpermeability and pulmonary edema [75]. Additionally, LPS reportedly downregulates ENaC-α expression via extracellular signal-regulated kinases 1/2 (ERK1/2) and p38 mitogen-activated protein kinase (MAPK) pathways [76], which may contribute to the impaired AFC of ALI/ARDS lungs. Moreover, in an LPS-induced ALI/ARDS model, LPS stimulates immune cells (e.g., macrophages and neutrophils) to produce different mediators, including cytokines, such as TNF-α and IL-1β, which downregulate ENaC-α expression and/or activity in alveolar epithelial cells and impair AFC capacity [77, 78]. Interestingly, in addition to mechanical stimulation, ENaC can also be activated by reactive oxygen species (ROS) [79], which are generally increased in ALI/ARDS lungs [80–82], For instance, in an LPS-induced ALI/ARDS model, LPS can stimulate ROS production by activating the NADPH oxidase 2/Rac1 pathway, and elevated ROS increased ENaC activity and enhanced AFC capacity [80], indicating that ROS-induced enhancement of ENaC activity may be a protective mechanism to counteract edema formation during the exudative phase of ALI/ARDS.

Furthermore, ENaC is also expressed in pulmonary microvessel endothelial cells. In LPS-treated mice, pulmonary endothelial permeability is markedly increased in mice with conditional knockout of ENaC-α in the endothelium (Endo-αENaC^KO^ mice) compared with control mice [83]. The stimulation of endothelial ENaC-α can attenuate pneumolysin-induced pulmonary endothelial hyperpermeability by blunting CaMKII activation and FLN-A phosphorylation, which suggests that endothelial ENaC may play a key role in protecting the endothelial barrier function in the presence of bacterial toxins (e.g., LPS and pneumolysin) [34, 84]. Notably, ENaC is reportedly involved in the pathogenesis of coronavirus disease (COVID-19)-mediated ALI/ARDS, and severe acute respiratory syndrome coronavirus (SARS-CoV), including SARS-CoV-1E, SARS-CoV-2E and SARS-CoV-2S proteins, markedly inhibits ENaC currents [85]. Additionally, SARS-CoV-2 spike protein contains an 8-mer peptide that is identical to the furin cleavage site of ENaC-α, and proteolytic cleavage of the furin site of ENaC is essential for its activation [86], SARS-CoV-2 infection may inhibit ENaC activity by hijacking the proteolysis network of ENaC, subsequently affect the AFC capacity and pulmonary edema of COVID-19 patient lungs [87].

**Fig. 4:**
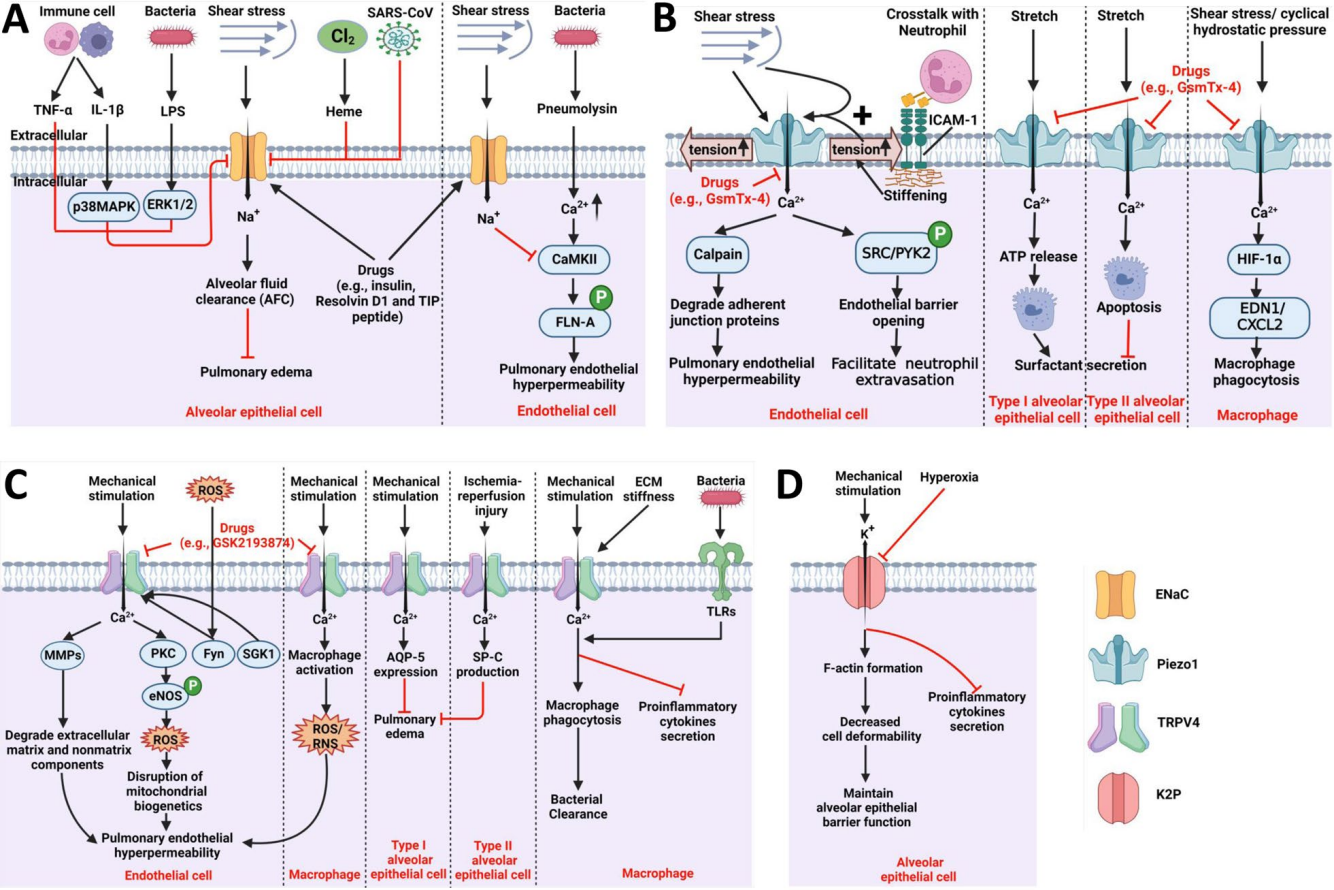
Cellular and molecular mechanisms of mechanosensitive ion channel-mediated mechanotransduction in acute lung injury/acute respiratory distress syndrome (ALI/ARDS). **A–D** Roles and mechanisms of ENaC (**A**), Piezo1 (**B**), TRPV4 (**C**) and K2P channels (**D**) in regulating alveolar fluid clearance, alveolar epithelial/endothelial barrier function, inflammatory response and surfactant secretion of ALI/ARDS. *ENaC* epithelial sodium channels, *TRPV4* transient receptor potential vanilloid 4, *K2P channels* two-pore domain potassium channels, *TNF-α* tumor necrosis factor-α, *IL-1β* interleukin-1β, *p38MAPK* p38 mitogen-activated protein kinase, *ERK1/2* extracellular signal-related kinase 1 and 2, *CaMKII* calmodulin-dependent protein kinase II, *FLN-A* filamin A, *ICAM-1* intercellular adhesion molecule 1, *SRC/PYK2* sarcoma/protein tyrosine kinase 2, *HIF-1α* hypoxia-inducible factor 1α, *EDN1/CXCL2* endothelin 1/CXL motif chemokine ligand 2, *MMPs* matrix metalloproteinases, *PKC* protein kinase c, *eNOS* endothelial nitric oxide synthase, *ROS/RNS* reactive oxygen/nitrogen species, *SGK1* serum glucocorticoid regulated kinase1, *AQP-5* aquaporin-5, *SP-C* surfactant protein c, *ECM* extracellular matrix, *TLRs* toll like receptors (figure created using BioRender.com)

Additionally, in multiple (e.g., LPS-induced) ALI/ARDS models, various drugs (e.g., insulin and resolvin D1) have been proven to efficiently alleviate pulmonary edema and attenuate lung injury by upregulating ENaC expression and/or activity [88, 89]. Moreover, the lectin-like domain of TNF, a TIP peptide that can enhance ENaC activity by binding to the ENaC-α subunit, improves the AFC capacity and alleviates pulmonary edema in pneumolysin-, listeriolysin O-*,* and ventilator-induced ALI/ARDS models [34, 84, 90–92]. The TIP peptide was recently shown to exert potential therapeutic effects on the treatment of pulmonary edema in ALI/ARDS patients. In a randomized, double-blind, placebo-controlled clinical trial (NCT01627613), TIP peptide (AP301) inhalation attenuated pulmonary edema in mechanically ventilated patients with severe ALI/ARDS (SOFA score ≥ 11) but failed to reduce pulmonary edema in patients with mild to severe ALI/ARDS (SOFA score ≤ 10) [93]. Additional multicenter clinical trials are needed to further clarify the therapeutic effect of TIP peptide inhalation. Taken together, the findings indicate that ENaC is a promising therapeutic target for the treatment of various types of ALI/ARDS (summarized in Table 1 and Fig. 4A）.

### Piezo channels in ALI/ARDS

Since their discovery in 2010 [94], Piezo channels (Piezo1 and Piezo2) have been increasingly recognized as one of the most important families of MSCs. Piezo channels are inherently MSCs that can sense mechanical forces through changes in their curvature (the force-from-lipids model) [17, 41]. Mechanical force-induced membrane tension can open Piezo channels to allow the influx of cations (Ca^2+^  > Na^+^  = K^+^) [50, 95], which can alter the membrane potential and activate downstream signaling pathways. Piezo channels are ubiquitously expressed in the human body and act as key mediators of various mechanotransduction processes, such as touch, blood flow, epithelial homeostasis, and cardiovascular homeostasis [26, 95–97]. In the lungs, the Piezo1 channel is highly expressed in pulmonary microvessel endothelial cells and alveolar epithelial cells [29, 53], and the Piezo2 channel is highly expressed in airway-innervating sensory neurons [28]. Recently, the emerging role of Piezo channels in the pathophysiology of ALI/ARDS has gradually become clearer (summarized in Table 1 and Fig. 4B).

Alveolar-capillary barrier dysfunction is an important pathological hallmark of ALI/ARDS, and both sterile (e.g., ventilator-induced stretch) and infectious (e.g., sepsis) causes of ALI/ARDS can result in injury to pulmonary microvascular endothelial cells [1, 3]. Pulmonary endothelial hyperpermeability results in the leakage of protein-rich edema fluid and immune cell infiltration. Most recent studies have indicated that the endothelial Piezo1 channel plays a deleterious role in regulating pulmonary endothelial barrier function [49, 98, 99]. Shear stress created by elevated blood flow or stretch force transmitted from alveoli can activate endothelial Piezo1 channels. Once endothelial Piezo1 channels are activated, Piezo1 channel-mediated Ca^2+^ influx can activate the protease calpain and subsequently degrade endothelial adherent junction proteins (e.g., VE-cadherin), thus causing pulmonary endothelial hyperpermeability [99]. Endothelium-specifical deletion of Piezo1 (Piezo1^iEC−/−^) in mice results in pulmonary endothelial hyperpermeability and pulmonary edema, which may contribute to the reduced lung static compliance in Piezo1^iEC−/−^ mice similar to the decreased lung compliance observed in ALI/ARDS patients [50]. Pulmonary endothelial hyperpermeability and pulmonary edema induced by high-volume ventilation or increased pulmonary microvessel pressure are abrogated in endothelial-specific deletion of Piezo1 (Piezo1^iEC−/−^) or Piezo1-knockdown mice [29, 99], and treatment with a Piezo1 inhibitor (GsmTx-4) can attenuates lung vascular leakage and edema formation [99]. However, another study found that endothelial Piezo1 is beneficial for maintaining adherens junctions and alveolar-capillary barrier function. As demonstrated with a ventilator-induced ALI/ARDS model, the expression levels of Piezo1 and VE-cadherin are decreased in mouse and patient lung samples undergoing long-term ventilation, and Piezo1 activation by cycle stretch enhances calpain activity, which suppresses Src-mediated VE-cadherin phosphorylation and subsequently stabilizes VE-cadherin junctions. Deletion of endothelial Piezo1 induces endothelial hyperpermeability and aggravates ventilator-induced ALI/ARDS [50], but the reason for the conflicting results needs further investigation.

Moreover, numerous studies have found that relative surfactant deficiency/dysfunction contributes to the lung surface tension distribution and reduced lung compliance of ALI/ARDS patients, and surfactant therapy may be a promising therapeutic strategy for ALI/ARDS [1, 3]. Lung surfactant is secreted by alveolar epithelial type II cells, and mechanical deformation/expansion of the alveoli is the strongest stimulus for surfactant secretion. A recent study found that Piezo1 channels may play a key role in surfactant secretion. Mechanical stretching can activate the Piezo1 channel in alveolar epithelial type I cells to trigger ATP release and subsequent paracrine stimulation of surfactant secretion from alveolar epithelial type II cells [53]. Additionally, in a ventilator-induced ALI/ARDS model, the Piezo1 channel expressed in alveolar epithelial type II cells can also be activated by mechanical stretch, and Piezo1-mediated Ca^2+^ influx induces apoptosis of alveolar epithelial type II cells, which may aggravate the surfactant deficiency during ALI/ARDS [54]. Further evidence is needed to confirm the relationship between the epithelial Piezo1 channel and ALI/ARDS.

Furthermore, infiltration of immune cells in the lungs has been recognized as another important pathological hallmark of ALI/ARDS [3, 5]. Piezo1 expressed in immune cells was recently proven to play a crucial role in the pathophysiology of ALI/ARDS [45, 100]. In response to bacterial infection, macrophages extravasate through the endothelium and engulf the bacteria, and macrophages exposed to shear stress are provoked by extravasation and cyclical hydrostatic pressure in the lungs. Piezo1-mediated mechanotransduction in alveolar macrophages is essential for physiological protection against bacterial infection. Alveolar macrophages, not neutrophils, isolated from the lungs of steady-state and *P. aeruginosa-*infected mice exhibit highly expression of Piezo1 channels. Piezo1-mediated mechanotransduction is needed for alveolar macrophages to respond to cyclical hydrostatic pressure with hypoxia-induced factor 1α (HIF1α) stabilization and secretion of molecules, such as endothelin 1 (EDN1) and neutrophil chemoattractant CXCL2. After infection with *P. aeruginosa*, higher bacterial loads, fewer tissue-infiltrating neutrophils (not macrophages) and decreased levels of inflammatory mediators have been found in the lungs of mice with macrophage-specific deletion of Piezo1 (Piezo1^ΔLysM^) than in their wild-type littermates, which suggests that Piezo1-mediated mechanotransduction in alveolar macrophages may play an anti-inflammatory role in infectious-associated ALI/ARDS [45]. Additionally, endothelial Piezo1-mediated mechanotransduction is needed for polymorphonuclear leukocyte (PMN, also called neutrophils) extravasation during inflammation. As demonstrated with the LPS-induced ALI/ARDS model, decreased neutrophil extravasation has been observed in the lungs of Piezo1^iEC−/−^ mice. Neutrophil-induced clustering of intercellular adhesion molecule-1 (ICAM-1) and fluid shear stress synergize to mechanically activate endothelial Piezo1, and Piezo1-mediated Ca^2+^ influx subsequently activates downstream signaling events (e.g., phosphorylation of SRC and PYK2) and stimulates neutrophil extravasation [100].

## TRP channels in ALI/ARDS

The transient receptor potential (TRP) superfamily of nonselective cation channels is divided into seven subfamilies: TRPV, TRPC, TRPA, TRPM, TRPML, TRPP/PKD, and TRPN/NompC [17]. The majority of TRP channels show mechanosensitive characteristics and have been implicated in various mechanosensation/mechanotransduction processes, including nociception, cancer metastasis, and control of the vascular tone [101–103]. Among these TRPs, TRPN/NomPC was the first definitively confirmed as a bona fide MSC tether gated through the microtubule cytoskeleton (the force-from-tethers model) [21, 104]. Mammalian TRPs, particularly TRPV4, are directly and efficiently activated by pilus-deflection mechanical stimulation (not stretch) [105, 106], and emerging data indicate that the tethering of TRPs (e.g., TRPV4) to the nonmembrane component enables TRP mechanosensitivity (the force-from-tethers model) [40, 105]. TRPs are involved in sensing a wide range of stimuli, including physical (mechanical stimulation, heat, cell swelling and pH) [107] and chemical stimuli (reactive oxygen and nitrogen species), and can be activated by LPS and elevated oxidative stress during the development of ALI/ARDS [60, 108, 109]. TRPs perform their effector functions primarily via Ca^2+^ influx currents. Accumulating evidence indicates that TRPs are key regulators and integrators of several major features of ARDS, including mechanosensing and mechanotransduction, redox sensing, the inflammatory response, and alveolar epithelial and endothelial barrier function [31, 42, 60, 110].

Of all TRPs, TRPV4 has attracted the most attention as a promising therapeutic target against ALI/ARDS (summarized in Table 1 and Fig. 4C). TRPV4 is abundantly expressed in lung tissues (epithelial and endothelial cells, macrophages, and neutrophils) and has been implicated in several animal models of ALI/ARDS [31, 42]. The role of TRPV4 in ALI/ARDS is dependent on the context/etiology. Numerous studies indicate that TRPV4 exerts a deleterious effect on the development of sterile or noninfectious stimulus-induced ALI/ARDS [33, 37]. In ventilator-induced ALI/ARDS, TRPV4 expressed in pulmonary microvessel endothelial cells can be activated by mechanical stimulation. TRPV4-mediated Ca^2+^ influx enhances PKC-dependent eNOS phosphorylation, promotes eNOS uncoupling, increases ROS production, disrupts mitochondrial bioenergetics, and degrades extracellular matrix and nonmatrix components (e.g., integrins and VE-cadherins) by activating of matrix metalloprotein 2 and 9 (MMP2 and 9), which ultimately results in endothelial hyperpermeability [111, 112]. Moreover, during the development of ALI/ARDS, elevated oxidative stress (e.g., ROS) can also activate TRPV4 through Src family kinase (Fyn)-dependent endothelial barrier disruption [33, 60]. Additionally, TRPV4 expressed in alveolar macrophages plays an adverse role in the development of ventilator-induced ALI/ARDS, and macrophage TRPV4 can be activated by mechanical stimulation, which promotes macrophage activation, elevates ROS and RNS production, and subsequently increases endothelial permeability [7]. Accumulating evidence indicates that ventilator-induced ALI/ARDS can be alleviated by treatment with TRPV4 inhibitors (e.g., HC-067047 and GSK2193874) or in TRPV4^−/−^ mice [7, 33, 55, 107]. In chemically induced ALI/ARDS, these chemical stimuli (hydrochloric acid and chlorine gas) can increase endogenous TRP channel agonists (e.g., *N-*acylamides), which can activate TRPV4, and both the knockout of TRPV4 and treatment with TRPV4 inhibitors (e.g., GSK2220691 and GSK2337429A) can decrease immune cell infiltration, reduce oxidative mediator production and inflammatory cytokine release, prevent epithelial and endothelial barrier function, and improve lung function [37, 113]. However, TRPV4 expressed in alveolar epithelial cells plays a beneficial role in preventing pulmonary edema formation caused by ischemia- reperfusion (IR)-induced lung injury. Aggravated IR-induced edema is observed in the lungs of TRPV4^−/−^ mice compared to WT mice, and ablation of TRPV4 in alveolar epithelial type I cells decreases aquaporin-5 (AQP-5) expression at the plasma membrane, reduces cell migration and disrupts barrier function. Alveolar epithelial type II cells of TRPV4^−/−^ mice showed decreased production of prosurfactant protein C, which also exacerbates edema formation [31].

Emerging data indicate that TRPV4, particularly the alveolar macrophage TRPV4, plays a beneficial role in the initiation and development of nonsterile or infectious-associated (e.g., sepsis, LPS) ALI/ARDS [43]. For instance, TRPV4 exerts a protective effect in an experimental ALI/ARDS model of *Pseudomonas aeruginosa* pneumonia [43]. Phagocytosis of alveolar macrophages is essential for bacterial and particle clearance, resolution of inflammation and tissue remodeling of infectious-associated ALI/ARDS [42, 114]. Numerous studies have shown that both sterile and infectious triggers of ALI/ARDS result in lung ECM stiffness (reduced lung compliance). The ECM stiffness in the lung parenchyma is significantly augmented (> 8–25 kPa) in the inflamed or fibrotic lungs of ALI/ARDS patients compared with that of normal lungs (1–3 kPa) [46, 62]. Both bacterial toxins (e.g., LPS) and matrix stiffness can directly/indirectly activate TRPV4 and significantly stimulate macrophage phagocytosis. Deletion of TRPV4 can abrogate the LPS effect and the matrix stiffness effect on the phagocytosis process [42], indicating that LPS and matrix stiffness can stimulate macrophage phagocytosis in a TRPV4-dependent manner. Furthermore, activation of macrophage TRPV4 can exert an anti-inflammatory effect (e.g., ↓IL-1β and ↑IL-10) to counteract the LPS-induced pro-inflammatory response, and the TRPV4-mediated anti-inflammatory profile is dependent on the pathophysiological-range ECM stiffness [42]. Additionally, macrophage TRPV4 reportedly increases dual-specificity phosphatase 1 (DUSP1) and then mediates MAPK switching from JNK to p38 activation in a stiffness-dependent manner, which can enhance bacterial clearance and decrease pro-inflammatory cytokine secretion and thereby mitigate the pathogenesis of infectious-associated ALI/ARDS [43].

In addition to TRPV4 channels, other TRPs have been implicated in the pathogenesis of ALI/ARDS. TRPA1 and TRPV1 expressed in lung tissues can be activated by LPS, LPS can upregulate the expression of TRPA1 and TRPV1. TRPA1/TRPV1-mediated Ca^2+^ influx can promote ROS production, activate MAPK/NF-κB signaling, increase pro-inflammatory mediators and aggravate the inflammatory response in ALI/ARDS [115]. In endothelial cells, TRPC1-mediated Ca^2+^ influx can inhibit sphingosine kinase 1 (SPHK) activity and decrease sphingosine-1-phosphate (S1P) generation, which results in disruption of adherens junctions and induction of endothelial hyperpermeability [116]. Furthermore, in IR-induced or infectious-associated ALI/ARDS, elevated oxidative stress (e.g., ROS) or LPS/TLR4 signaling can increase diacylglycerol (DAG) production, which directly activates endothelial TRPC6, and augmented TRPC6-mediated Ca^2+^ influx also results in endothelial hyperpermeability and pulmonary edema [109, 117]. In macrophages, redox-sensitive TRPM2 can be activated by elevated ROS, and TRPM2-mediated Ca^2+^ influx can negatively regulate ROS production and decrease pro-inflammatory cytokine and chemokine release, and endotoxin-induced lung injury is exacerbated in TRPM2^−/−^ mice [118].

## K2P channels in ALI/ARDS

The two-pore domain potassium ion channels (K2P/KCNK) form background (leak) K^+^ selective channels and conduct outward K^+^ currents that result in resting membrane potential hyperpolarization [119]. Among the K2P channels, three members of the K2P family, including TREK-1 (K2P2.1/KCNK2), TREK-2 (K2P10.1/KCNK10), and TRAAK (K2P4.1/KCNK2), are inherently MSCs [22, 120]. These three channels are sensitive to a wide range of tensions, from 0.5 mN/m to the membrane lytic point of 12 mN/m, and can directly sense mechanical forces applied to the lipid membrane (force-from-lipids model) [22, 121], and mice lacking these three genes exhibit hypersensitivity to mechanical stimuli [122]. These three channels exhibit polymodal gating by various mechanical stimuli (stretching, cell swelling and poking), heat, pH, chemical stimuli (e.g., arachidonic acid), and drugs (e.g., volatile anesthetics, antidepressants) [123–125]. These three K2P channels are highly expressed in lung tissues, including alveolar epithelial cells and macrophages [36]. The expression/activity of these K2P channels can be affected by inflammatory cytokines (e.g., TNF-α) or life-saving therapies (e.g., mechanical ventilation and hyperoxia) during the development of ALI/ARDS, and these K2P channels, particularly the TREK-1 channel, are suggested to act as key regulators of alveolar epithelial barrier function and the inflammatory response in ALI/ARDS (summarized in Table 1 and Fig. 4D) [8, 36].

As an inherent MSC, the TREK-1 channel regulates the mechanobiology of alveolar epithelial cells. In cultured alveolar epithelial cells, ablation of TREK-1 results in less F-actin formation but increased cell deformability, which exerts a protective effect on cyclic stretch-induced cell detachment and may subsequently affect alveolar epithelial barrier function [126]. More in vivo evidence is needed to evaluate the role of TREK-1 in ventilator-induced ALI/ARDS. Emerging evidence indicates that TREK-1 also acts as a “regulatory molecule” in the inflammatory response and cell proliferation of ALI/ARDS [127, 128]. TNF-α is the main pro-inflammatory cytokine in the bronchoalveolar lavage fluid of ALI/ARDS patients. In vitro, ablation of TREK-1 alters the cytokine release profile of alveolar epithelial cells upon TNF-α stimulation (e.g., ↓IL-6 and ↑MCP-1) and increases the proliferation of alveolar epithelial cells. Additionally, TREK-1 is suggested to play a more prominent role in hyperoxia than ventilator-induced ALI/ARDS [8]. Hyperoxia exposure can decrease TREK-1 expression (not TREK-2 or TRAAK) in mouse lungs and cultured alveolar epithelial cells and inhibit TREK-1-stimulated cell proliferation [36]. As observed in an in vivo ALI/ARDS model, hyperoxia exposure further exacerbates the lung injury of TREK-1^−/−^ mice compared with WT mice, as demonstrate by decreased lung compliance, increased lung injury scores, promotion of immune cell infiltration and activation of the proapoptotic signaling pathway, but the alveolar-capillary barrier function is not affected (no increase in BAL protein) [129]. Intratracheal administration of TREK-1 activators can alleviate hyperoxia-induced ALI/ARDS [8]. Similarly, exacerbated lung injury associated with decreased surfactant protein A and C (SPA and SPC) has also observed in TREK-1/TREK-2/TRAAK triple knockout (TKO) mice compared with WT mice, which suggests that TREK-2 and TRAAK may also be involved in the development of hyperoxia-induced ALI/ARDS [32]. The detailed molecular and cellular mechanisms underlying K2P channels in ALI/ARDS require further investigation.

## Outlook and conclusion

Substantial progress over the past decades has greatly expanded our knowledge about the structure and function of MSCs and of the role and underlying mechanisms of MSCs in the pathogenesis of ALI/ARDS (summarized in Table 1 and Fig. 4). However, our current understanding of the relationship between MSCs and ALI/ARDS remains largely unknown, and there are several gaps in the knowledge of MSCs and ALI/ARDS: (1) Accumulating evidence indicates that the activities of different types of MSCs can be mutually regulated, which may be involved in several pathological conditions and diseases. For instance, in human umbilical vein endothelial cells (HUVECs), activation of Piezo1 by high shear stress or agonists can initiate an elevation in intracellular Ca^2+^ ([Ca^2+^]_i_) and stimulate the activation of phospholipase A2 (PLA2), which facilitates TRPV4 opening and in turn causes sustained [Ca^2+^]_i_ elevation to result in adherens junction disruption and cytoskeletal remodeling [130]. Because various MSCs are expressed in the same cell types in lung tissues, further investigations are needed to determine how cells integrate different mechanical inputs during ALI/ARDS and the mechanism that regulates the balance between depolarization and hyperpolarization MSCs. (2) In addition to these aforementioned MSCs, whether other well-recognized MSCs that are expressed in other mechanosensory cells (e.g., transmembrane channel 1/2 (TMC1/2) expressed in inner ear hair cells) and new candidates for MSCs (e.g., TACAN and Elkin1) [23, 131, 132] are involved in the pathogenesis of ALI/ARDS needs further investigation. (3) Regardless of the gating mechanism of MSCs, all MSCs are embedded in a membrane bilayer and subjected to the mechanical properties of the surrounding lipids, particularly cholesterol-rich lipid rafts. Recent studies have indicated that many members of the stomatin protein family, including stomatin, stomatin-like protein-3 (STOML3) and MEC-2, are enriched in cholesterol-rich lipid rafts and can affect the mechanosensitivity of MSCs [133–135]. For instance, STOML3 can modulate membrane mechanics by binding to cholesterol, which results in promoting of force transfer and regulating the mechanosensitivities of MSCs, including Piezo channels [133]. Therefore, additional studies are needed to determine whether these membrane stiffness regulators also tune the mechanosensitivities of MSCs in lung tissues and thereby affect the development of ALI/ARDS. (4) Most studies were performed using in vitro cultured cells and in vivo animal models, and the difference between animal ALI/ARDS models and human ALI/ARDS patients should be taken into consideration. Further work is needed to explore the changes in the expression and/or activities of MSCs in the lung tissues of patients with ALI/ARDS. Moreover, with the development of more specific MSC agonists/antagonists, additional high-quality clinical trials are needed to confirm the therapeutic effects of these drugs in human ALI/ARDS patients.

**Table 1:** In vitro and in vivo studies the roles of mechanosensitive ion channels (MSCs) in ALI/ARDS

Channels	Cell types	Key findings	References
ENaC	Alveolar epithelial cells	Promotes alveolar fluid clearance and attenuates pulmonary edema	[75, 77, 78]
	Endothelial cells	Protects endothelial barrier function	[83, 84]
Piezo1	Alveolar epithelial cells	Triggers alveolar type I cells ATP release and paracrine stimulation of surfactant secretion from alveolar type II cells	[53]
		Induces alveolar type II cells apoptosis	[54]
	Endothelial cells	Activation of Piezo1 disrupts endothelial adherens junction proteins and causes endothelial hyperpermeability	[29, 49]
		Protects adherens junction and matains endothelial barrier function	[50]
		Endothelial Piezo1-mediated Ca^2+^ influx stimulates neutrophil extravasation	[100]
	Macrophages	Enhances macrophage phagocytosis and promotes bacterial clearance	[45]
TRPV4	Alveolar epithelial cells	Activation of TRPV4 disrupts epithelial barrier function	[37, 112]
		Maintains epithelial barrier function	[31]
	Endothelial cells	Activation of TRPV4 disrupts endothelial barrier function	[111, 112]
	Macrophages	Lipopolysaccharide/matrix stiffness-induced macrophage phagocytosis in vitro and in vivo	[7, 42]
		Anti-inflammatory cytokine production (IL-1β, IL-10)	
TRPV1/TRPA1	Immune cells (macrophages, neutrophils)	Elevates ROS production,activates MAPK/NF-κB signaling, increases proinflammatory mediators and aggravates inflammatory response	[115]
TRPC1	Endothelial cells	Disrupts adherens junction and induces endothelial hyperpermeability	[116]
TRPC6	Endothelial cells	Induces endothelial hyperpeamability	[109, 117]
TRPM2	Macrophages	Decreases the ROS production and reduces pro-inflammatory cytokine and chemokine release	[118]
TREK-1	Alveolar epithelial cells	Decreases cell deformability and inhibit stretch-induced cell detachment	[126]
		Stimulates cell proliferation	[127]
		Anti-inflammatory cytokine production (↓IL-6 and ↑MCP-1)	[127, 128]
		Hyperoxia exposure decreases the TREK-1 expression	[36]

*MSCs* mechanosensitive ion channels, *ENaC* epithelial sodium channel, *TRPV 1/4* transient receptor potential vanilloid ¼, *TRPA1* transient receptor potential ankyrin 1, *TRPC1/6* transient receptor potential canonical 1, *TRPM2* transient receptor potential melastatin 2, *TREK-1* TWIK-related potassium channel 1

In summary, emerging data indicate that mechanical forces and other stimuli can modulate the activities of MSCs in lung tissues, and dysfunction of MSCs is associated with the development of ALI/ARDS. MSCs play a key role in regulating alveolar fluid clearance, alveolar epithelial/endothelial barrier function, inflammatory response and surfactant secretion in ALI/ARDS lungs. Targeting MSCs appears to be a potential novel therapeutic strategy for the treatment of ALI/ARDS.

## Data Availability

Not applicable.
